# Biological and Pharmacological Activities of Squalene and Related Compounds: Potential Uses in Cosmetic Dermatology

**DOI:** 10.3390/molecules14010540

**Published:** 2009-01-23

**Authors:** Zih-Rou Huang, Yin-Ku Lin, Jia-You Fang

**Affiliations:** 1 Pharmaceutics Laboratory, Graduate Institute of Natural Products, Chang Gung University, 259 Wen-Hwa 1^st^ Road, Kweishan, Taoyuan 333, Taiwan; 2 Graduate Institute of Clinical Medical Sciences, Chang Gung University, Kweishan, Taoyuan, Taiwan; 3 Department of Traditional Chinese Medicine, Chang Gung Memorial Hospital, Keelung, Taiwan

**Keywords:** Squalene, skin surface lipid, skin, antioxidant, topical delivery

## Abstract

Squalene is a triterpene that is an intermediate in the cholesterol biosynthesis pathway. It was so named because of its occurrence in shark liver oil, which contains large quantities and is considered its richest source. However, it is widely distributed in nature, with reasonable amounts found in olive oil, palm oil, wheat-germ oil, amaranth oil, and rice bran oil. Squalene, the main component of skin surface polyunsaturated lipids, shows some advantages for the skin as an emollient and antioxidant, and for hydration and its antitumor activities. It is also used as a material in topically applied vehicles such as lipid emulsions and nanostructured lipid carriers (NLCs). Substances related to squalene, including β-carotene, coenzyme Q10 (ubiquinone) and vitamins A, E, and K, are also included in this review article to introduce their benefits to skin physiology. We summarize investigations performed in previous reports from both *in vitro* and *in vivo* models.

## Introduction

**Figure 1:**
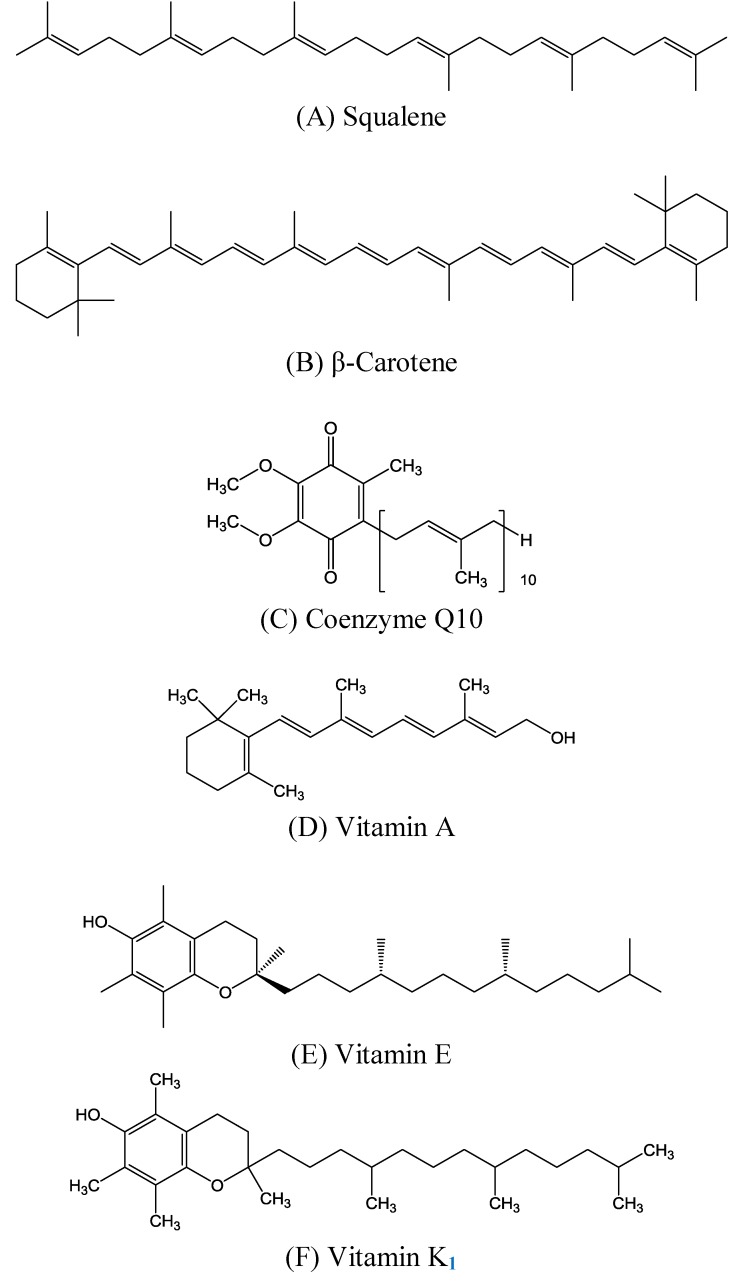
Chemical structures of (A) squalene, (B) β-carotene, (C) coenzyme Q10, and (D) vitamins A, (E) E, and (F) K_1_.

Human skin, covering the entire outer surface of the body, is the largest organ and is constantly exposed to sunlight stress, including ultraviolet (UV) light irradiation. The skin tissue is rich in lipids, which are thought to be vulnerable to oxidative stress from sunlight. Squalene (Figure 1A) is a structurally unique triterpene compound that is one of the main components (about 13%) of skin surface lipids [1]. It was so named because it was first isolated from shark (*Squalus* spp.) liver oil, which contains large quantities and is considered its richest source [2]. It is transported in serum generally in association with very low density lipoproteins and is distributed ubiquitously in human tissues, with the greatest concentration in the skin.

Experimental studies have shown that squalene can effectively inhibit chemically induced skin, colon, and lung tumorigenesis in rodents [3]. The protective effect is observed when squalene is given before and/or during carcinogen treatment. The mechanisms involved in the chemopreventive activity of squalene may include inhibition of Ras farnesylation, modulation of carcinogen activation, and antioxidative activities [4]. However, several factors must be considered when the evidence for the inhibition of carcinogenesis by squalene is examined. These include the effective dose used and the time of exposure [5]. This type of information is obtained from animal bioassays, and the long-term effects of consuming increased levels of squalene are not known. Although animal studies have enhanced our understanding of the possible actions of squalene in decreasing carcinogenesis, one must apply caution in extrapolating the information obtained in animal studies to humans, because of possible differences among species.

Many other polyprenyl compounds structurally similar to squalene exist in nature and perform critical biological functions. These include β-carotene (Figure 1B), coenzyme Q10 (Figure 1C), and vitamins A (Figure 1D), E (Figure 1E), and K_1_ (Figure 1F), which are introduced here because of their benefits to skin physiology. For example, animals utilize prenyl groups to form the side chain of coenzyme Q10. The coenzyme Q10 designation indicates that the molecule has 10 prenyl groups in its side-chain. Other well-known substances require prenyl groups for their synthesis, and therefore are structurally similar to squalene. In the present work, we attempted to introduce the activities and benefits of squalene and related molecules to skin tissue, the largest organ of the human body. Some important *in vivo* and clinical studies of squalene and its derivatives are also summarized and reviewed in this article.

## Biological activities of squalene

Squalene appears to be critical for reducing free radical oxidative damage to the skin. Serum squalene originates partly from endogenous cholesterol synthesis and partly from dietary sources, especially in populations consuming large amounts of olive oil or shark liver [2]. The endogenous synthesis of squalene begins with the production of 3-hydroxy-3-methylglutaryl coenzyme A (HMG CoA). The initial reduction of HMG CoA (a niacin-dependent reaction) results in the formation of mevalonate [4].

**Figure 2:**
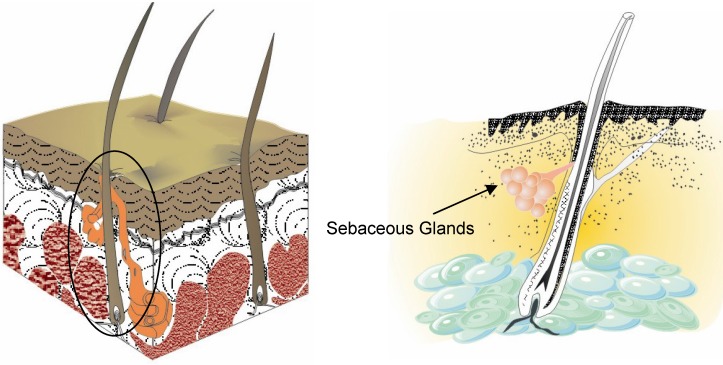
Sectional view of the skin with sebaceous glands.

Sebaceous glands are small glands in the skin which secrete an oily matter (sebum) in the hair follicles to lubricate the skin and hair of animals (Figure 2). In humans, they are found in the greatest abundance on the face and scalp, although they are distributed throughout all skin sites except the palms and soles. Squalene is one of the predominant components (about 13%) of sebum (Table 1) [5].

**Table 1:** Composition of sebum in humans.

Substance	Composition (%)
Wax esters	25
Squalene	13
Cholesterol	2
Triglycerides, free fatty acids, and diglycerides	57
Other components	3

## Effects of squalene on the skin

Squalene is not very susceptible to peroxidation and appears to function in the skin as a quencher of singlet oxygen, protecting human skin surfaces from lipid peroxidation due to exposure to UV light and other sources of oxidative damage [6], as discussed here.

### Emollient

Truly one of nature’s great emollients, squalene is quickly and efficiently absorbed deep into the skin, restoring healthy suppleness and flexibility without leaving an oily residue. New cosmetic emulsions with biomimetic molecules have been investigated using experimental designs [7]. That study determined the optimal composition of a squalene mixture in an oil-in-water emulsion, using a design of experiments to elaborate the experimental strategy. For this purpose, the stability, centrifugation, viscosity, and pH of squalene were measured, and a microscopic analysis was carried out. Results showed that the stability and viscosity of the emulsions exhibited the greatest influence on the percentage of squalene.

### Skin hydration

In general, occlusion leads to increased skin hydration due to reduced water loss. Rissmann *et al.* [8] revealed that a *vernix caseosa* (VC) substitute can be an innovative barrier cream for barrier-deficient skin. This is because of the excellent properties of VC in facilitating stratum corneum hydration. Different lipid fractions were isolated from lanolin and subsequently mixed with squalene, triglycerides, cholesterol, ceramides, and fatty acids to generate semi-synthetic lipid mixtures that mimic the lipid composition of VC. The results showed that the rate of barrier recovery increased and was comparable to VC lipid treatment. Okuda *et al.* [9] also found that the elevated transepidermal water loss (TEWL) and riboflavin penetration in 5% sodium lauryl sulfate-treated rat and human skin were reversed by squalene supplementation (*p* < 0.05).

### Antioxidation

Squalene has been reported to possess antioxidant properties. *In vitro* experimental evidence indicates that squalene is a highly effective oxygen-scavenging agent. Subsequent to oxidative stress such as sunlight exposure, squalene functions as an efficient quencher of singlet oxygen and prevents the corresponding lipid peroxidation at the human skin surface [10]. Kohno *et al.* [11] found that the rate constant for quenching singlet oxygen by squalene is much larger than those of other lipids on the human skin surface, and was comparable to that of 3,5-di-*t*-butyl-4-hydroxytoluene. They also reported that squalene is not particularly susceptible to peroxidation and is stable against attacks by peroxide radicals, suggesting that the chain reaction of lipid peroxidation is unlikely to be propagated with adequate levels of squalene present on the human skin surface. Aioi *et al.* [12] studied the effects of squalene on superoxide anion (O_2_^-^) generation in rats in order to elucidate the mechanism whereby this compound decreases erythema induced by 1% lauroylsarcosine (LS) ointment. LS (200~400 μg/mL) caused overt production of O_2_^-^ from cultured keratinocytes and peritoneal exudate leukocytes. O_2_^-^ was significantly reduced by the addition of squalene (100 μg/mL). These results suggest that a possible role of squalene for alleviating skin irritation is by suppression of O_2_^-^ production, which is dependent on different mechanisms of action of superoxide dismutase.

### Antitumor activities

During the past few years, squalene was found to show protective activities against several carcinogens [13]. Desai *et al.* [14] reported that skin tumors were initiated in 50 female CD-l mice with 7,12-dimethylbenz[a]anthracene and promoted with 12-*O*-tetradecanoylphorbol-13-acetate. The mice were treated with 5% squalene and at the end of the prevention study, there was a 26.67% reduction in the incidence of tumors in the squalene-treated group. In a related branch of research, a protective effect was observed when squalene was given before and/or during carcinogen treatment. Experimental studies have shown that squalene can effectively inhibit chemically induced skin tumorigenesis in rodents [15].

## Squalene as a material in topical formulations

Squalene is also used as a material or additive in topically applied vehicles such as lipid emulsions and nanostructured lipid carriers (NLCs).

### Lipid emulsions

**Figure 3:**
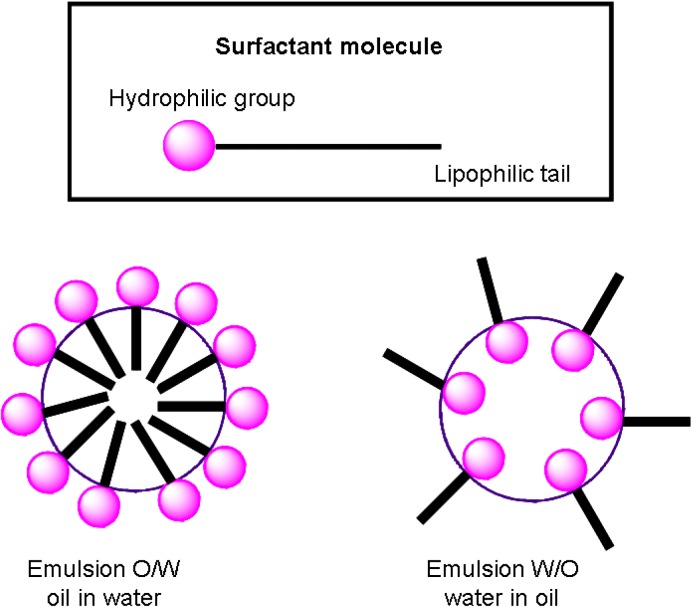
A potent drug carrier vehicle: lipid emulsions.

Lipid emulsions are potentially interesting drug delivery systems because of their ability to incorporate drugs with poor solubility within the dispersal phase (Figure 3). An emulsion is a mixture of two immiscible (unblendable) liquids. Lipid emulsions have been studied as parenteral drug carriers for sustained release and organ targeting. By using lipid emulsions, direct contact of the drug with the body fluid and tissues can also be avoided to minimize possible side effects [16]. Chung *et al.* [17] prepared oil-in-water type lipid emulsions to investigate the effects of different oils on emulsion particle size and stability. Squalene was shown to form stable emulsions when a lipophilic drug was loaded in the discontinuous oil phase. Even though the *in vitro* transfection activity of emulsions was lower than that of liposomes in the absence of serum, the activity of squalene emulsions, for instance, was approximately 30 times higher than that of liposome in the presence of 80% (v/v) serum (*p* < 0.05).

Kim *et al.* [18] found that a squalene emulsion had the most potent transfection activity and showed the least cytotoxicity in a mouse model after intravenous administration. Squalene as the oil component can enhance the stability of cationic emulsions more effectively which might be useful for *in vitro* and *in vivo* gene transfer. In addition, Wang *et al.* [19] indicated that emulsions with squalene as the oil phase can act as a potential parenteral drug delivery system for nalbupline and its prodrugs. Squalene as the oil phase produced the smallest particle size compared to coconut oil. The *in vivo* analgesic activity of the emulsions was examined by a cold ethanol tail-flick test. The squalene system showed the ability to provide controlled delivery to prolong the analgesic duration in rats. The toxicity determined by erythrocyte hemolysis was also low for squalene emulsions.

### Nanostructured lipid carriers (NLCs)

**Figure 4:**
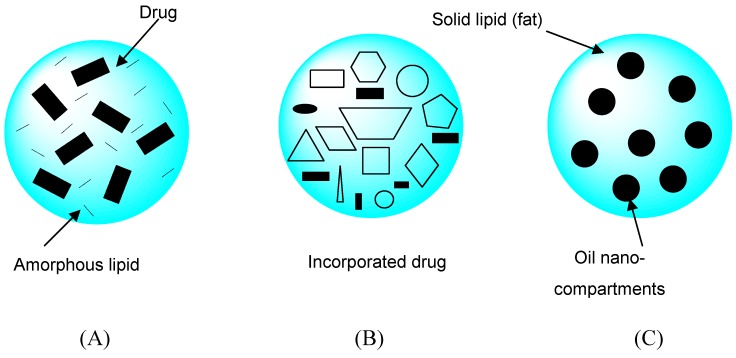
Three different types of nanostructured lipid carriers (NLCs) compared to the more or less highly ordered matrix of solid lipid nanoparticles (SLNs). The three types of NLC can be summarized as: (A) the amorphous type, (B) imperfect type, and (C) multiple type. This figure is modified from reference [24].

Solid lipid nanoparticles (SLNs) are a new generation of oil-in-water nanoparticulate systems and are attracting attention as novel colloidal drug carriers. Distinct advantages of SLNs are the solid state of the particle matrix, the ability to protect chemically labile ingredients, and the possibility of modulating and prolonging drug release. NLCs are a novel type of lipid nanoparticle with a solid particle matrix possessing structural specialties and improvements such as an increased loading capacity, long-term physical and chemical stability, triggered release, and potentially supersaturated topical formulations. NLCs are produced by mixing solid lipids with spatially incompatible lipids leading to a lipid matrix with a special structure. Depending on the method of production and composition of the lipid blend, different types of NLCs can be obtained (Figure 4). The basic idea is that by giving the lipid matrix a certain nanostructure, the payload of active compounds is increased and expulsion of the compound during storage is avoided [20,21,22,23,24].

Fang *et al.* [25] found that NLCs consisting of Precirol^®^ and squalene (12% w/v) showed respective mean particle sizes of 200 nm. The lipophilicity of NLCs decreased with an increase in the squalene content in the formulations. Psoralen derivatives (i.e. 8-methoxypsoralen) for psoriasis treatment were loaded in NLCs to examine their ability to permeate via the skin. Enhanced permeation and controlled release of psoralen were both achieved using NLCs with squalene. The *in vitro* permeation results showed that NLCs stabilized with Tween^®^ 80 increased the 8-methoxypsoralen flux 2.8 times over that of a conventional emulsion.

## Production of toxins by squalene oxidation

Squalene monohydroperoxide (SQOOH) is a primary oxidized lipid produced from squalene by solar UV. It is produced at the human skin surface due to natural exposure to sunlight during daily activities. Recent studies demonstrated that repeated application of SQOOH to the skin can induce skin roughness and wrinkles in the hairless mouse [26]. Uchino *et al.* [27] also reported that SQOOH induced skin damage in hairless mice. Furthermore, a few papers have also described the cytotoxicity of SQOOH, which is a primary oxidized form of epidermal lipids produced by solar UV rays. SQOOH is produced on human forehead skin, and it was suggested that skin squalene may be the principal target lipid for oxidative stress (e.g., sunlight exposure). Nakagawa *et al.* [28] demonstrated that SQOOH accumulation may be involved in inflammatory skin disorders such as skin cancer, cutaneous autoimmune disease, and skin aging.

Chiba *et al.* [29] studied the effects of SQOOH, i.e., the initial product of UV-peroxidated squalene, on the skin of hairless mice. Repeated topical application of 10 mM SQOOH to hairless mice for 15 weeks induced definite skin wrinkling. Those results clearly suggested that wrinkling and changes in dermal collagen content induced by SQOOH qualitatively differ from those induced by UVB exposure (*p* < 0.001). This may provide a useful model for studying skin aging, particularly with regard to the collagen content.

## Biological activities of squalene analogs

### β-Carotene

Many other polyprenyl compounds structurally similar to squalene exist in nature and perform critical biological functions. β-Carotene is well known to be a potent ^1^O_2_ quencher and unique free radical scavenger [30]. This pro-vitamin A, carotenoid, can accumulate in the human skin through oral intake. Stahl *et al.* [31] demonstrated that oral supplementation of carotenoid from sea algae *Dunaliella salina* (94% β-carotene and small amounts of α-carotene, cryptoxanthin, zeaxanthin, and lutein) protected human skin from UV light-induced erythema. Bando *et al.* [30] found that dietary β-carotene acts as a photoprotective agent in the skin. That *in vivo* study focused on determining the mechanism of action of β-carotene against UVA-induced skin damage by characterizing β-carotene oxidation products. BALB/c mice were fed a β-carotene-supplemented diet, and homogenates from the dorsal skin were prepared after three weeks for UVA irradiation. The results indicated that dietary β-carotene accumulated in the skin and acted as a protective agent against UVA-induced oxidative damage, by quenching ^1^O_2_.

A number of studies have demonstrated that dietary β-carotene protects human skin from UV light-induced erythema, but little is known about the protective effect of dietary β-carotene on UVA-induced skin photoaging [32]. Antille *et al.* [33] showed that topical β-carotene penetrated well into the human and mouse epidermis and induced 10- (human) and a 3-fold (mouse) increases, respectively, in epidermal retinyl esters, which demonstrates that topical β-carotene is converted into retinyl esters by human and mouse epidermis and thus appears as a precursor of epidermal vitamin A.

β-Carotene was partially successful in treating a photosensitivity disorder, erythropoietic protoporphyria, of which singlet oxygen is believed to be an important mediator. Several studies were performed to examine whether β-carotene protects against UV-induced erythema in healthy humans, with widely differing reported effects. The incidence of nonmelanoma skin cancer was reported to be inversely related to serum β-carotene concentrations, and earlier experimental UV-carcinogenesis studies found β-carotene to be photoprotective [34]. However, the role of β-carotene as an anticancer agent was questioned as a result of randomized intervention studies in which supplementation did not reduce the incidence of nonmelanoma skin cancers in humans. Some concern exists over the long-term (eight weeks) use of high-dose β-carotene because supplements of this substance can have deleterious effects at higher doses (15 mg/day) in human skin (*p* < 0.05).

### Coenzyme Q10

Coenzyme Q10 is an important lipophilic antioxidant synthesized by the body [34]. Topical coenzyme Q10 treatment may therefore be proposed as a good pharmacological tool in dermatology and cosmetology [35]. The amount of coenzyme Q10 in skin decreases with age. Topical application of coenzyme Q10 to human skin was found to be effective in reducing the depth of wrinkles. Coenzyme Q10 (0, 1, and 100 mg/kg oral administration) was administered daily for two weeks. Supplementation with 100 mg/kg coenzyme Q10 significantly increased serum and epidermal coenzyme Q10 levels, but did not increase coenzyme Q10 levels in the dermis or other organs. This indicates that coenzyme Q10 intake elevates the epidermal coenzyme Q10 level, which may be a prerequisite to reducing wrinkles and for other benefits related to the potent antioxidant and energizing effects of coenzyme Q10 in the skin [36].

Coenzyme Q10 is a popular antioxidant used in many skin care products to protect the skin from free radical damage. The effects of coenzyme Q10 and colorless carotenoids on the production of inflammatory mediators in human dermal fibroblasts treated with UV radiation and possible synergistic effects of these two antioxidants were evaluated by Fuller *et al.* [36]. Treatment of fibroblasts with 10 µM coenzyme Q10 suppressed the UV- and interleukin (IL)-1-induced increases in prostaglandin E2, IL-6, and matrix metalloproteinase (MMP)-1. The results suggested that the combination of carotenoids and coenzyme Q10 in topical skin care products may provide enhanced protection from inflammation and premature aging caused by sun exposure.

### Vitamin A

Squalene and some of its related substances, including vitamin A, were examined in an animal model to determine the existence of chemopreventive effects. Varani *et al.* [37] pointed out that topical vitamin A (retinol) stimulated new collagen deposition in sun-protected aged skin, as it did in photoaged skin. In a separate group of 53 individuals (80 years of age), topical application of 1% vitamin A for seven days increased fibroblast growth and collagen synthesis, and concomitantly reduced the levels of matrix-degrading MMPs. In addition, vitamin A treatment reduced MMP expression in aged, sun-protected skin. Retinyl esters, a storage form of vitamin A, are concentrated in the epidermis and absorb UV radiation with a maximum at 325 nm. Antille *et al.* [38] applied topical retinyl palmitate onto the back of hairless mice before exposing them to 1 J/cm^2^ UVB, and assayed the levels of thymine dimers produced in epidermal DNA 2 h following UVB exposure. The results demonstrated that epidermal retinyl esters have a biologically relevant filter activity and suggest, besides their pleomorphic biologic actions, a new role for vitamin A that is concentrated in the epidermis (*p* < 0.05). Moreover, Alberts *et al.* [39] concluded that vitamin A doses of 50,000 and 75,000 IU/day for one year proved safe, were equally more efficacious than a 25,000 IU/day dose, and could be recommended for future skin cancer chemoprevention studies.

Vitamin A-derived agents still continue to be used to treat acne. Furthermore, retinoids act as chemopreventive and/or chemotherapeutic agents for several types of cancer. They have major effects on the growth and differentiation of normal, premalignant, and malignant epithelial cells both *in vitro* and *in vivo* [40]. Retinol (vitamin A) is widely used in the cosmetics industry as an anti-wrinkle agent. However, its photoinstability and skin irritation potential make it challenging to use in general cosmetic formulations [41]. Retinol was applied to skin in either an oil-in-water emulsion or a gel vehicle. Because of the substantial skin reservoir for retinol (0.3%) found at the end of 24 h in human skin studies, additional studies were conducted to compare the *in vitro* and *in vivo* skin absorption levels of retinol in the fuzzy rat. Results from those studies were used to help interpret the significance of the *in vitro* retinol human skin reservoir in determining systemic absorption (*p* < 0.001) [42]. Jenning *et al.* [43] evaluated the potential use of solid lipid nanoparticles (SLNs) in dermatology and cosmetics. The influence on drug penetration into porcine skin of glyceryl behenate SLNs loaded with vitamin A (retinol and retinyl palmitate) and incorporated in a hydrogel and oil-in-water cream was tested. Excised full-thickness skin was mounted in an *in vitro* Franz diffusion assembly, and formulations were applied for 6 and 24 h. Vitamin A concentrations in the skin tissue suggested a certain drug localizing effect. High retinol concentrations were found in the upper skin layers following application of SLN preparations, whereas the deeper regions showed only very low vitamin A levels (*p* < 0.05).

### Vitamin E

Vitamin E is the most potent lipid-soluble antioxidant *in vivo*. It is thought to play an important role in skin protection [44]. Uddin *et al.* [45] examined the protective efficacy of vitamin E against tumors induced by UV and arsenite. Hairless mice were exposed to UV plus sodium arsenite (5 mg/L in drinking water) and fed laboratory chow supplemented with vitamin E (*R,R,R*-*a*-tocopheryl acetate, 62.5 IU/kg diet) for 26 weeks. Vitamin E reduced the tumor yield in mice given UV and arsenite by 2.1-fold (*p* < 0.001). Those results show that vitamin E can strongly protect against arsenite-induced enhancement of UV-caused carcinogenesis.

Vitamin E is a group of eight different compounds, but only two of the forms, α-tocopherol and γ-tocopherol, are commonly found in the human body [44]. Lopez-Torres *et al.* [46] investigated the effects of topical α-tocopherol application on epidermal and dermal tissues and its ability to prevent UV-induced oxidative damage. Hairless mice received a topical application of α-tocopherol 24 h before a single acute exposure to UV irradiation (10 × minimal erythemal dose). α-Tocopherol treatment significantly reduced the formation of epidermal lipid hydroperoxides after UV irradiation (*p* < 0.05). Those results demonstrated that topical administration of α-tocopherol protects cutaneous tissues against oxidative damage induced by UV irradiation *in vivo*.

Yoshida *et al.* [47] investigated whether the topical application of a novel, water-soluble γ-tocopherol derivative, γ-tocopherol-*N,N*-dimethylglycinate hydrochloride (γ-TDMG), could protect against UV-induced skin damage in hairless mice. Topical pre- or post-application of a 5% (93 mM) γ-TDMG solution in water/propylene glycol/ethanol (2:1:2) significantly prevented sunburned cell formation, lipid peroxidation, and edema/inflammation, which were induced by exposure to a single dose of UV irradiation at 5 kJ/m^2^ (290~380 nm, maximum 312 nm). Those results suggest that the topical application of γ-TDMG may be efficacious in preventing and reducing UV-induced inflammation.

Ekanayake-Mudiyanselage *et al.* [48] recently demonstrated that even the use of rinsing products containing α-tocopherol in concentrations of < 0.2% can lead to significantly increased levels of vitamin E in the stratum corneum of human skin and protects against lipid peroxidation *in vivo*. Therefore, topical formulations containing α-tocopherol at concentrations ranging from 0.1% to 1% are likely to be effective skin care measures to enhance antioxidative protection of the skin barrier. According to the antioxidant network theory, combinations with co-antioxidants such as vitamin C may help enhance the antioxidant effects and stability of vitamin E [49]. A better knowledge of the unique skin-specific physiology of vitamin E, including its percutaneous penetration, skin barrier interactions, bioconversion of vitamin E esters, and cutaneous delivery pathways of oral vitamin E could help in developing more-efficacious skin care products and better evaluating indications and dosage regimens for preventing and treating acute and chronic skin disorders.

### Vitamin K

Vitamin K is another squalene-related substance that exhibits benefits to skin physiology. Lou *et al.* [50] examined the safety and efficacy of a topical vitamin K cream for shortening the duration of laser-induced purpura. From the results of that work, a combination of 1% vitamin K and 0.3% retinol in an acrylate copolymer cream hastened the resolution of laser-induced purpura.

The application of vitamin K to the skin has also been used to suppress pigmentation and resolve bruising. Lopes *et al.* [51] investigated the *in vitro* skin penetration and transdermal delivery of vitamin K (2.5%, w/w), and whether these parameters were enhanced by lipid-based drug delivery systems. The experimental results demonstrated that the topical delivery of vitamin K incorporated in a lipophilic vehicle was low. It could be enhanced (~3-fold increase) by monoolein-based systems, which may be useful in increasing the effectiveness of topical vitamin K therapy.

## Conclusions

Squalene appears to be critical in reducing free radical oxidative damage to the skin. Although epidemiological, experimental, and animal evidence suggests antitumor properties, few human trials have been conducted to date to verify the role of squalene in cancer therapy. Further studies are needed to explore the usefulness of squalene for treating skin. Several implications can be drawn from this review. Squalene shows several advantages for skin tissues. It is also useful as a material in topically applied vehicles. Substances related to squalene such as β-carotene, coenzyme Q10, and vitamins A, E, and K also exhibit their benefits for skin physiology. Topical administration via the skin is an important route to supplement these compounds within skin tissues. The present success of squalene and its analogs shows the promise of further clinical trials for skin use.
